# A new contingent screening strategy increased detection rate of trisomy 21 in the first trimester

**DOI:** 10.1186/s12884-023-06115-1

**Published:** 2023-11-14

**Authors:** Wei Luo, Bin He, Daiwen Han, Lixing Yuan, Jun Tang, Ling Pang, Fene Zou, Kai Zhao, Shanling Liu, Ting Hu

**Affiliations:** 1 Department of Medical Genetics, West China Second University Hospital, Sichuan University, Chengdu, Sichuan 610041 China; 2 Department of Obstetrics and Gynecology, West China Second University Hospital, Sichuan University, Chengdu, Sichuan 610041 China; 3 Key Laboratory of Birth Defects and Related Diseases of Women and Children (Sichuan University), Ministry of Education, Chengdu, Sichuan 610041 China

**Keywords:** Trisomy 21, Serum biochemical screening, First-trimester screening, Non-invasive prenatal screening, Prenatal diagnosis, Contingent screening strategy

## Abstract

**Background:**

Although the traditional contingent screening strategy is effective, there are still undetected low-risk trisomy 21. This study aims to define appropriate cut-off values of serum biochemical markers at low-risk and develop a strategy for sequential prenatal testing associated with first-trimester screening to increase the detection rate of trisomy 21.

**Methods:**

This was a 9-year retrospective analysis of singleton pregnant women who underwent serum biochemical screening or combined first-trimester screening (CFTS) in the first trimester. For the low-risk group, the cut-off values of the serum biochemical markers were adjusted to determine the appropriate detection efficiency. Gravidas with abnormal serum biochemical markers at low-risk were advised to undergo further non-invasive prenatal screening (NIPS), whereas others continued with routine prenatal care.

**Results:**

When cut-off values of free beta subunit of human chorionic gonadotropin (free β-hCG) multiples of the median (MoM) or pregnancy-associated plasma protein A (PAPP-A) MoM were defined with ≥ 2.75 or ≤ 0.5, 7.72% (2,194/28,405) in the serum biochemical screening group and 12.36% (4,005/32,403) in CFTS group could be detected as abnormal results for further NIPS. Finally, 55.56% (5/9) and 85.71% (6/7) of trisomy 21 cases with false-negative results were detected, and the overall detection rate for trisomy 21 was improved by 10.64% (5/47) and 12.77% (6/47), respectively.

**Conclusions:**

The new contingent screening strategy can increase the detection rate of trisomy 21 compared with the traditional contingent screening strategy.

## Backgrounds

Trisomy 21, also known as Down Syndrome (DS), is the most common chromosomal aneuploid abnormality, with a prevalence in live birth of approximately 1/700 in developed countries and 1/1,100 ~ 1/750 in developing countries [1, 2]. Patients with DS are characterized by facial features, neuromotor delay, hypotonia, heart defects, deafness, ocular problems, and a higher risk of malignancies [2, 3], causing serious mental and economic burdens to families and society. Although there are several treatment methods, such as logopedic and motor therapy, heart corrections, cochlear implants, none of them can cure the syndrome fundamentally, only improve the quality of life. During the gestation period, the confirmation of trisomy 21 allows parents to decide whether they want or not to continue the pregnancy. The main method is prenatal aneuploidy screening, which can be performed for all gravidas if they want. For those at high-risk, invasive prenatal diagnoses are sequentially performed via amniocentesis or foetal blood sampling [1, 4].

Currently, the main methods of prenatal aneuploidy screening are serological screening, non-invasive prenatal screening (NIPS), and ultrasonographic screening. Serum biochemical markers combined with nuchal translucency (NT) measurements have long been the mainstay of foetal aneuploidy screening in the first trimester. In the past decade, NIPS, which is based on massively parallel genomic sequencing technology, has been widely applied for the clinical detection of trisomy 21, trisomy 18, and trisomy 13 [5]. Many studies have indicated the excellent performance of NIPS in the prenatal screening of common aneuploidies [6–9]. Although the NIPS has been suggested as a first-tier screening tool, it is difficult to promote its price in developing countries. Recently, a series of studies showed a cost-effective contingent screening strategy in which high-, intermediate-, and low-risk groups were stratified through traditional screening methods and underwent invasive testing, NIPS, and no further testing. The contingent screening strategy implied an improvement in the trisomic detection rate and a reduction in healthcare costs [10–14].

According to *Technical standards of prenatal screening and diagnosis for foetal common chromosomal abnormalities and open neural tube defects Part1 Maternal serum prenatal screening in the second trimester (the Health Standards of the People’s Republic of China, 2010)* and *Technical Specification for Prenatal Screening and Diagnosis of NIPS (National Health Commission of the People’s Republic of China, 2016)*, in China, women of advanced maternal age were offered the option of amniocentesis and other women underwent serum biochemical screening or combined first-trimester screening (CFTS) as the first-tier test. The traditional contingent screening strategy was widely used in China as follows, according to the results of first-tier screening, women with a high-risk (≥ 1:270) were offered invasive prenatal diagnosis, those with an intermediate-risk (1:1,000 ~ 1:271) were offered NIPS, and those with low-risk (< 1:1,000) were suggested no further testing. Although this contingent screening strategy was better than NIPS as a first-tier screening in terms of health economy, there are still undetected low-risk trisomy 21.

Our research aimed to define appropriate cut-off values of serum biochemical markers at low-risk and develop a strategy for sequential prenatal diagnosis associated with first-trimester screening to increase the detection rate of trisomy 21.

## Materials and methods

### Study population

A total of 41,612 singleton pregnancies and 41,270 singleton pregnancies undergoing serum biochemical screening and CFTS for common fetal chromosomal abnormalities at the Prenatal Diagnosis Center of West China Second University Hospital were recruited between January 2011 and December 2019. The study was approved by the Institutional Ethics Committee of Sichuan University, and all participants provided written informed consent prior to the test. The research was conducted in accordance with relevant guidelines and clinical norms. Professional counselling was offered by trained clinical geneticists before undergoing antenatal test for chromosomal and genetic abnormalities detection, which aimed to prevent misunderstandings with potential medico legal consequences [15]. The inclusion criteria were as follows: (1) gravidas with maternal age ≥ 16 years, and (2) pregnancy gestation period between 11 weeks and 13^+ 6^ weeks calculated according to crown-rump length in the first trimester. The exclusion criteria were as follows:1) gravidas with a family history of chromosomal abnormalities, 2) foetuses with structural malformations, (3) multiple pregnancies or co-twin demise, (4) gravidas with the termination of pregnancy (TOP) or stillbirth without chromosomal results, and 4) loss of follow-up assessments.

### Clinical follow-up assessments

We recorded all pregnancy outcomes, including miscarriage, TOP, and delivery. Foetal chromosomes were confirmed by karyotyping, chromosomal microarray analysis, copy number variation sequencing of amniotic fluid samples by prenatal diagnosis, and foetal tissue sampling in cases without delivery. For live births, clinical follow-up assessments were conducted via medical record review or telephone calls six months after the expected date of confinement.

### Screening method

The NT refers to the fluid-filled space in the dorsal aspect of the foetal neck [1]. A transabdominal ultrasound examination was performed to obtain a sagittal section of the foetus to measure the crown-rump length and the maximum thickness of the subcutaneous translucency between the skin and the soft tissue overlying the cervical spine.

The free beta subunit of human chorionic gonadotropin (free β-hCG) and pregnancy-associated plasma protein A (PAPP-A) was detected according to the manufacturer’s instructions (Perkin Elmer, USA). The risk of serum biochemical screening and CFTS was calculated using the Lifecycle software (Perkin Elmer, USA).

The risk of serum biochemical screening was calculated based on two serum markers, free β-hCG and PAPP-A, combined with maternal age. The risk of CFTS was calculated based on the measurements of NT and two serum markers, free-hCG and PAPP-A, combined with maternal age [16].

Biochemical markers and NT thickness measurements were converted into multiples of the median (MoM) for gestational age and adjusted for maternal weight, insulin-dependent diabetes mellitus, smoking status, and race. The MoM values were centre specific. The risk of trisomy 21 was estimated by multiplying the maternal age-specific odds of live births of an infant affected by trisomy 21 with the likelihood ratio obtained from the overlapping Gaussian distributions of affected and unaffected pregnancies [16].

### Study design

This was a retrospective analysis of singleton pregnancies who underwent serum biochemical screening or CFTS in the first trimester. We collected the results of serum biochemical screening or CFTS and pregnancy outcomes and analysed the performance of the two screening tests as first-tier tests. Gravidas were divided into three groups for trisomy 21: high-risk (≥ 1:270), intermediate-risk (1:1,000 ~ 1:271), and low-risk (< 1:1,000). The high-risk group underwent invasive tests, and the intermediate-risk group underwent NIPS. For those gavidas with positive NIPS results, invasive prenatal diagnosis was subsequently performed subsequently to confirm the percision of NIPT. For the low-risk group, the cut-off values of the serum biochemical markers were adjusted to determine the appropriate detection efficiency. Gravidas with abnormal serum biochemical markers at low-risk were advised to undergo consecutive NIPS, whereas others continued with routine prenatal care. Furthermore, we compared the clinical performance of this new contingent screening strategy with that of the traditional contingent screening strategy. On this basis, it was assumed that the detection rate and false-positive rate of NIPS were consistent with those reported, with a detection rate of 99% and a false-positive rate of 0.5% [17]. The uptake rates of NIPS and invasive prenatal diagnosis were assumed to be 100%.

### Statistical analysis

The statistical software package SPSS 23.0 (SPSS Inc., Chicago, IL, USA) was used for the data analyses. Descriptive data were presented as median (interquartile range (IQR)) for continuous variables and n (%) for categorical variables. Prenatal screening algorithm performance measures were presented as the detection rate and odds of being affected given a positive result (OAPR). Comparisons between the groups were performed using the Wilcoxon rank-sum test for categorical variables.

## Results

### Characteristics of the study population

After excluding 6,304 in the serum biochemical screening group and 6,244 in the CFTS group for loss to follow-up or TOP without foetal karyotype, 35,308 and 35,026 singleton pregnancies, respectively, were recruited in our study. There were no significant differences in the age distribution, method of conception, maternal weight, gestational age, insulin-dependent diabetes, or smoking ratio between the serum biochemical screening and CFTS groups (Table 1). In both groups, the MoM of free-hCG in foetuses diagnosed with trisomy 21 was significantly higher than those unaffected (*P* < 0.01). In contrast, the MoM of PAPP-A was significantly lower (*P* < 0.01) (Table 2).

**Table 1:** Pregnancy characteristics of women with two screening tests

Characteristic	Serum biochemical screening	Combined first-trimester screening	*P*
**Number**	35,308	35,026	
**Age distribution**			
Maternal age at expected date of delivery < 35 years(%)	34,724(98.35%)	34,467(98.40%)	0.543
Maternal age at expected date of delivery ≥ 35 years(%)	584(1.65%)	559(1.60%)	0.543
Maternal age (years) at expected date of delivery (IQR)	30.00(28.06–32.13)	30.02(27.52–31.62)	0.215
**Method of conception (%)**			
Spontaneous	33,118(93.80%)	32,798(93.64%)	0.387
Assisted	2,190(6.20%)	2,228(6.36%)	0.387
**Median maternal weight (IQR)**	53(49–58)	53(49–58)	0.896
**Median gestational age (days) at blood sample (IQR)**	88(85–92)	88(85–92)	0.605
**Insulin-dependent diabetes (%)**	24(0.07%)	24 (0.07%)	0.978
**Smoker (%)**	362(1.03%)	356(1.02%)	0.907
**Incidence of Down syndrome(‰)**	47(1.33‰,1/751)	47(1.34‰,1/745)	0.969

*Data are given as median (interquartile range) or n (%). IQR, interquartile range

**Table 2:** Characteristics of biomakers of singleton pregnancies affected by trisomy 21 and unaffected pregnancies

Characteristic	Serum biochemical screening			Combined first-trimester screening		
	Unaffected	Trisomy 21	*P*	Unaffected	Trisomy 21	*P*
Number	35,261	47		35,979	47	
Serum free β-hCG (MoM)	0.99(0.67 ~ 1.52)	2.14(1.21 ~ 2.93)	< 0.01	0.99(0.67 ~ 1.52)	2.31(1.23 ~ 2.94)	< 0.01
Serum PAPP-A (MoM)	1.02(0.71 ~ 1.43)	0.42(0.25 ~ 0.60)	< 0.01	1.02(0.71 ~ 1.43)	0.42(0.26 ~ 0.62)	< 0.01

Data are given as median (interquartile range) or n (%). β-hCG, beta-human chorionic gonadotropin, PAPP-A, pregnancy-associated plasma protein-A. MoM,multiples of the median

### Performance of screening for trisomy 21 by traditional contingent screening strategy

In the serum biochemical screening group, 1,874 (5.31%), 5,029 (14.24%), and 28,405 (80.45%) patients were classified as high-, intermediate-, and low-risk, respectively. Additionally, in the CFTS group, 875 (2.50%), 1,748 (4.99%), and 32,403 (92.51%) patients were classified as high-, intermediate-, and low-risk, respectively (Table 3).

**Table 3:** Performance of two screening tests with traditional contingent screening strategy

Risk stratification	Serum biochemical screening			Combined first-trimester screening		
	n(%)	Trisomy 21 (%)	OAPR	n(%)	Trisomy 21 (%)	OAPR
high risk (risk ≥ 1/270)	1,874 (5.31%)	29 (61.70%)	1:64	875 (2.50%)	34 (72.34%)	1:26
intermediate risk (1/1,000 ≤ risk < 1/270)	5,029 (14.24%)	9 (19.15%)	1:558	1,748 (4.99%)	6 (12.77%)	1:291
low risk (risk < 1/1,000)	28,405 (80.45%)	9 (19.15%)		32,403 (92.51%)	7 (14.89%)	

OAPR: Odds of being affected given a positive result

In the serum biochemical screening group, the proportion of high- and intermediate-risk was 19.55% (6,903/35,308), while the proportion of trisomy 21 diagnoses was 80.85% (38/47). The OAPR of high- and intermediate-risk patients were 1:64 and 1:558, respectively. In the CFTS group, the proportion of high- and intermediate-risk patients was 7.49% (2,623/35,026), whereas the proportion of trisomy 21 diagnoses was 85.11% (40/47). The OAPR of high- and intermediate-risk patients were 1:26 and 1:291, respectively (Table 3).

In the serum biochemical screening group, the proportion of low-risk patients was 80.45% (28,405/35,308), and trisomy 21 with false-negative results was 19.15% (9/47) However, in the CFTS group, the proportion of low-risk patients was 92.51% (32,403/35,026), and trisomy 21 with false-negative results was 14.89% (7/47) (Table 3).

### Performance of screening for trisomy 21 by new contingent screening strategy

To define appropriate cut-off values of serum biochemical markers in low-risk, free β-hCG was increased with 0.25 MoM per layer from 2.00 to 3.00 MoM and PAPP-A with 0.1 MoM per layer from 0.1 to 0.8 MoM in serum biochemical screening and CFTS group, respectively (Tables 4 and 5).

**Table 4:** Performance of different serum marker MoM in serum biochemical screening

Serum marker		Abnormal serum marker*		Trisomy 21**		OAPR
free β-hCG MoM	PAPP-A MoM	n	%	n	%	
2.00	0.10	1,505	4.26	2	4.26	1:753
2.00	0.20	1,533	4.34	2	4.26	1:767
2.00	0.30	1,704	4.83	2	4.26	1:852
2.00	0.40	2,252	6.38	3	6.38	1:751
2.00	0.50	3,337	9.45	5	10.64	1:667
2.00	0.60	4,885	13.84	5	10.64	1:977
2.00	0.70	6,811	19.29	7	14.89	1:973
2.00	0.80	9,061	25.66	8	17.02	1:1,133
2.25	0.10	891	2.52	2	4.26	1:446
2.25	0.20	919	2.60	2	4.26	1:460
2.25	0.30	1,090	3.09	2	4.26	1:545
2.25	0.40	1,638	4.64	3	6.38	1:546
2.25	0.50	2,723	7.71	5	10.64	1:545
2.25	0.60	4,271	12.10	5	10.64	1:854
2.25	0.70	6,197	17.55	7	14.89	1:885
2.25	0.80	8,447	23.92	8	17.02	1:1,056
2.50	0.10	561	1.59	2	4.26	1:281
2.50	0.20	589	1.67	2	4.26	1:295
2.50	0.30	760	2.15	2	4.26	1:380
2.50	0.40	1,308	3.70	3	6.38	1:436
2.50	0.50	2,393	6.78	5	10.64	1:479
2.50	0.60	3,941	11.16	5	10.64	1:788
2.50	0.70	5,867	16.62	7	14.89	1:838
2.50	0.80	8,117	22.99	8	17.02	1:1,015
2.75	0.10	362	1.03	2	4.26	1:181
2.75	0.20	390	1.10	2	4.26	1:195
2.75	0.30	561	1.59	2	4.26	1:281
2.75	0.40	1,109	3.14	3	6.38	1:370
2.75	0.50	2,194	6.21	5	10.64	1:439
2.75	0.60	3,742	10.60	5	10.64	1:748
2.75	0.70	5,668	16.05	7	14.89	1:810
2.75	0.80	7,918	22.43	8	17.02	1:990
3.00	0.10	221	0.63	0	0.00	-
3.00	0.20	249	0.71	0	0.00	-
3.00	0.30	420	1.19	0	0.00	-
3.00	0.40	968	2.74	1	2.13	1:968
3.00	0.50	2,053	5.81	3	6.38	1:684
3.00	0.60	3,601	10.20	3	6.38	1:1,200
3.00	0.70	5,527	15.65	5	10.64	1:1,105
3.00	0.80	7,777	22.03	6	12.77	1:1,296

*the number of abnormal serum marker in low risk. **the number of trisomy 21 of abnormal serum marker in low risk

β-hCG, beta-human chorionic gonadotropin, PAPP-A, pregnancy-associated plasma protein-A, MoM,multiples of the median, OAPR: Odds of being affected given a positive result

**Table 5:** Performance of different serum marker MoM in combined first-trimester screening

Serum marker		Abnormal serum marker*		Trisomy 21**		OAPR
free β-hCG MoM	PAPP-A MoM	n	%	n	%	
2.00	0.10	3,537	10.10	1	2.13	1:3,537
2.00	0.20	3,597	10.27	2	4.26	1:1,799
2.00	0.30	3,889	11.10	3	6.38	1:1,296
2.00	0.40	4,726	13.49	4	8.51	1:1,182
2.00	0.50	6,271	17.90	6	12.77	1:1,045
2.00	0.60	8,215	23.45	6	12.77	1:1,369
2.00	0.70	10,525	30.05	6	12.77	1:1,754
2.00	0.80	12,995	37.10	6	12.77	1:2,166
2.25	0.10	2,432	6.94	1	2.13	1:2,432
2.25	0.20	2,492	7.11	2	4.26	1:1,246
2.25	0.30	2,784	7.95	3	6.38	1:928
2.25	0.40	3,625	10.35	4	8.51	1:906
2.25	0.50	5,183	14.80	6	12.77	1:864
2.25	0.60	7,168	20.46	6	12.77	1:1,195
2.25	0.70	9,534	27.22	6	12.77	1:1,589
2.25	0.80	12,074	34.47	6	12.77	1:2,012
2.50	0.10	1,724	4.92	1	2.13	1:1,724
2.50	0.20	1,784	5.09	2	4.26	1:892
2.50	0.30	2,076	5.93	3	6.38	1:692
2.50	0.40	2,919	8.33	4	8.51	1:730
2.50	0.50	4,484	12.80	6	12.77	1:747
2.50	0.60	6,489	18.53	6	12.77	1:1,082
2.50	0.70	8,886	25.37	6	12.77	1:1,481
2.50	0.80	11,462	32.72	6	12.77	1:1,910
2.75	0.10	1,243	3.55	1	2.13	1:1,243
2.75	0.20	1,303	3.72	2	4.26	1:652
2.75	0.30	1,595	4.55	3	6.38	1:532
2.75	0.40	2,439	6.96	4	8.51	1:610
2.75	0.50	4,005	11.43	6	12.77	1:668
2.75	0.60	6,022	17.19	6	12.77	1:1,004
2.75	0.70	8,439	24.09	6	12.77	1:1,407
2.75	0.80	11,045	31.53	6	12.77	1:1,841
3.00	0.10	869	2.48	0	0.00	-
3.00	0.20	929	2.65	1	2.13	1:929
3.00	0.30	1,221	3.49	2	4.26	1:611
3.00	0.40	2,064	5.89	3	6.38	1:688
3.00	0.50	3,631	10.37	5	10.64	1:726
3.00	0.60	5,652	16.14	5	10.64	1:1,130
3.00	0.70	8,088	23.09	5	10.64	1:1,618
3.00	0.80	10,710	30.58	5	10.64	1:2,142

*the number of abnormal serum marker in low risk. **the number of trisomy 21 of abnormal serum marker and low risk

β-hCG, beta-human chorionic gonadotropin, PAPP-A, pregnancy-associated plasma protein-A, MoM,multiples of the median, OAPR: Odds of being affected given a positive result

In the serum biochemical screening group, in order to achieve OAPR higher than that of intermediate-risk and obtain a higher detection rate of trisomy 21, the cut-off value of abnormal biomarker was defined as free β-hCG MoM ≥ 2.75 or PAPP-A MoM ≤ 0.5 (Table 4). Although the OAPR in the CFTS group was significantly higher than that in the serum biochemical screening group (1:291 vs. 1:558), the OAPR in the low-risk group was not higher than that in the intermediate-risk group (Table 3). In the CFTS group, in order to achieve a relatively higher detection rate of trisomy 21 combined with a relatively lower proportion of abnormal biomarkers in low-risk, the cut-off value of abnormal biomarker was defined as free β-hCG MoM ≥ 2.75 or PAPP-A MoM ≤ 0.5 (Table 5).

When the cut-off value of free β-hCG MoM ≥ 2.75 or PAPP-A MoM ≤ 0.5 were defined, abnormal serum biochemical markers with low-risk for further NIPS could be detected as 7.72% (2,194/28,405) in the serum biochemical screening group and 12.36% (4,005/32,403) with low-risk in CFTS group. Furthermore, 55.56% (5/9) and 85.71% (6/7) trisomy 21 with false-negative results were detected in the serum biochemical screening and CFTS groups, respectively (Table 6).

**Table 6:** Performance of abnormal biomarkers in low risk

Risk stratification (risk < 1/1000)	Serum biochemical screening		Combined first-trimester screening		
	n(%)	Trisomy 21 (%)	n(%)	Trisomy 21 (%)	
free β-hCG MoM ≥ 2.75 PAPP-A MoM ≤ 0.5	2,194 (7.72%)	5 (55.56%)	4,005 (12.36%)	6 (85.71%)	
free β-hCG MoM < 2.75 PAPP-A MoM > 0.5	26,211 (92.28%)	4 (44.44%)	28,398 (87.64%)	1 (14.29%)	

β-hCG, beta-human chorionic gonadotropin, PAPP-A, pregnancy-associated plasma protein-A, MoM,multiples of the median

According to the results of first-tier screening, the new contingent screening strategy divided gravidas into four groups for trisomy 21: high-risk (≥ 1:270), intermediate-risk (1:1,000 ~ 1:271), abnormal biomarkers (free β-hCG MoM ≥ 2.75 or PAPP-A MoM ≤ 0.5) in low-risk, and others (Table 7). Gravidas in the high-risk group were offered invasive prenatal diagnosis, those in the intermediate-risk and abnormal biomarker groups were offered NIPS, and the others continued with routine prenatal care. Through a new contingent screening strategy, the overall detection rates of trisomy 21 were 91.49% (43/47) and 97.87% (46/47) in the first-tier serum biochemical screening and CFTS, respectively. (Table 7). False-negative cases of trisomy 21 were 4 and 1 in the serum biochemical screening and CFTS, respectively. In the serum biochemical screening group, the proportion of abnormal biomarkers was 6.21% (2,194/35,308), whereas in trisomy 21 with false-negative results, the proportion of abnormal biomarkers was 10.64% (5/47). In the CFTS group, the proportion of abnormal biomarkers was 11.43% (4,005/35,026), whereas in trisomy 21 with false-negative results, the proportion of abnormal biomarkers was 12.77% (6/47).

**Table 7:** Performance of two screening tests with new contingent screening strategy

Risk stratification	Serum biochemical screening			Combined first-trimester screening		
	n(%)	Trisomy 21 (%)	OAPR	n(%)	Trisomy 21 (%)	OAPR
high risk (risk ≥ 1/270)	1,874 (5.31%)	29 (61.70%)	1:64	875 (2.50%)	34 (72.34%)	1:26
intermediate risk (1/1,000 ≤ risk < 1/270)	5,029 (14.24%)	9 (19.15%)	1:558	1,748 (4.99%)	6 (12.77%)	1:291
free β-hCG MoM ≥ 2.75 PAPP-A MoM ≤ 0.5 (risk < 1/1,000)	2,194 (6.21%)	5 (10.64%)	1:439	4,005 (11.43%)	6 (12.77%)	1:668
free β-hCG MoM < 2.75 PAPP-A MoM > 0.5 (risk < 1/1,000)	26,211 (74.24%)	4 (8.51%)		28,398 (81.08%)	1 (2.13%)	

β-hCG: beta-human chorionic gonadotropin, PAPP-A: pregnancy-associated plasma protein-A, MoM,multiples of the median, OAPR: Odds of being affected given a positive result

## Discussion

Our 9-year large-scale retrospective study showed that the median and interquartile range of PAPP-A MoM and free-hCG MoM in gravidas with and without trisomy 21 (Table 2) were consistent with the data reported by Nicolaides et al. [18]. In addition, patients with 61.70% (29/47) and 72.34% (34/47) trisomy 21 were found to be at high-risk through serum biochemical screening and CFTS as first-tier screening, respectively, which were consistent with previous reports [4].

In China, serological prenatal screening has been used as a first-tier screening method owing to its low cost. Besides invasive prenatal diagnosis for high-risk patients, benefiting from the high detection performance of NIPS for those with intermediate-risk [7, 9, 19], the detection rate of trisomy 21 was further improved through traditional contingent screening based on serum biochemical screening and CFTS. However, in our study, 19.15% (9/47) and 14.89% (7/47) of trisomy 21 cases were not detected by serum biochemical screening and CFTS, respectively.

Previous studies have shown the advantage of contingent screening and suggested optimal cut-off values for risk [10]. The adoption of contingent screening implies a greater detection rate of trisomy 21 and a reduction in healthcare costs [14]. Contingent screening using conventional combined and second-trimester screening tests is effective [20]. Other studies show that effective first-trimester screening for trisomy 21, with a detection rate of 98% and invasive testing rate < 0.5%, can be potentially achieved by contingent screening incorporating NIPS and NT, ductus venosus pulsatility index for veins, serum-free β-hCG, PAPP-A, placental growth factor and alpha-fetoprotein [18]. The more markers used in the first-tier screening, the higher the trisomy 21 that could be detected by contingent screening. However, the application of new markers results in higher costs, technical platforms, and personnel training, which are difficult to achieve and promote in developing countries with limited health and economic development.

Without adding new markers, we created a new contingent screening strategy by defining appropriate cut-off values for abnormal serum biochemical markers at low-risk. Based on the traditional screening strategy widely used in China, in trisomy 21, the MoM of free-hCG increased, and that of PAPP-A decreased. Our study demonstrated that when abnormal biomarkers were defined as free β-hCG MoM ≥ 2.75 or PAPP-A MoM ≤ 0.5 in both serum biochemical screening and CFTS group, the detection rate of trisomy 21 was optimal. In the serum biochemical screening and CFTS groups, the OAPR of the low-risk group with abnormal biochemical marker levels were 1:439 and 1:668, respectively. As these OAPRs were within the range of intermediate-risk (1:1,000~1:271), we recommended a low-risk group with abnormal biochemical markers for further NIPS. Among low-risk patients, 7.72% (2,194/28,405) in the serum biochemical screening group and 12.36% (4,005/32,403) in the CFTS group had abnormal NIPS results. Meanwhile, 55.56% (5/9) and 85.71% (6/7) trisomy 21 with false-negative results were detected. In other words, the overall detection rate for trisomy 21 improved by 10.64% (5/47) in the serum biochemical screening group and 12.77% (6/47) in the CFTS group using a new contingent screening strategy without adding new biochemical markers and technical platforms.

Regardless of whether traditional or new contingent screening is used, the screening efficiency of CFTS as a first-tier screening method is better than that of serum biochemical screening. This was based on the accurate measurement of the NT value. The CFTS is recommended as a first-tier screening method for institutions that can effectively carry out NT measurements. Only one case of trisomy 21 was missed through the new contingent screening strategy for CFTS as a first-tier test, and the comprehensive detection rate was 97.87% (46/47). Meticulous technique in NT imaging and measurement is essential for accurate risk assessment because under measurement by even 0.5 mm can reduce the test sensitivity by 18% [1]. When NT measurements cannot be performed, or effective quality control of NT measurements is lacking, serum biochemical screening can be used as a first-tier screening method through the new contingent screening strategy. The detection rate of the new contingent strategy has increased from 80.85% (38/47) of the original traditional contingent screening detection rate to 91.49% (43/47). This new contingent screening strategy for trisomy 21, with serum biochemical screening as a first-tier test, may be suitable for areas with limited healthcare and economic resources. This strategy improved the detection rate of trisomy 21 without increasing technical difficulty and workload.

Invasive prenatal diagnosis of those with abnormal biochemical markers can accidentally detect other chromosomal abnormalities, such as sex chromosome abnormalities and microdeletion/microduplications, which are also related to adverse pregnancy outcomes [4]. A large dataset study showed that for patients with PAPP-A and free-hCG levels below 0.2 MoM, the prevalence of atypical chromosomal abnormalities was 6.9% and 5.2%, respectively [21]. The risks of aneuploidy, spontaneous abortion, preterm delivery, and small-for-gestational-age newborns increased with decreasing PAPP-A [22]. PAPP-A is a valuable analyte for predicting the risk of adverse pregnancy outcomes, and women with low serum PAPP-A levels may benefit from closer surveillance [23]. In the first trimester, unexplained low PAPP-A (< 0.4 MoM) and/or low hCG (< 0.5 MoM) levels are associated with an increased frequency of adverse obstetrical outcomes [24]. First-trimester biomarkers of PAPP-A and free-hCG could also be used as tools for risk identification of preterm birth without extra effort and cost [25]. According to the above research, the new contingent screening strategy could potentially screen for common chromosomal aneuploidies while evaluating adverse pregnancy outcomes according to the PAPP-A MoM level.

To the best of our knowledge, this is the first study in China to assess a new contingent screening strategy for trisomy 21 using serum biochemical screening and CFTS as first-tier tests. This is because the whole nation could not be covered by only one screening method due to the different detection abilities and economic situations among different regions of China; therefore, our study offers clinicians insights to explore a novel approach.

Our study has some limitations. First, we only enrolled pregnant women who elected to undergo prenatal screening by serum biochemical screening or combined first-trimester screening as the first-tier test, which may lead to selection bias. Second, we excluded the gavidas who were loss of follow-up, which may lead to non-respondent bias. Third, for those pregnant women suffered from spontaneous miscarriages, no matter at low-risk or high-risk, most of which did not demand to perform genetic tests for the foetuses, which may lead to bias of detection rate and OAPR.

## Conclusions

Appropriate biomarker cut-off values can effectively detect trisomy 21 at low-risk in first-trimester screening. For the new contingent screening strategy for trisomy 21, abnormal serum biochemical markers at low-risk were advised to undergo further NIPS, which can increase the detection rate of trisomy 21 compared with the traditional contingent screening strategy.

## Data Availability

The data that support the findings of this study are available from the corresponding author upon reasonable request.

## Abbreviations

CFTS: combined first-trimester screening
NIPS: non-invasive prenatal screening
free β-hCG: free beta subunit of human chorionic gonadotropin
MoM: multiples of the median
PAPP-A: pregnancy-associated plasma protein A
DS: Down Syndrome
NT: nuchal translucency
TOP: termination of pregnancy
IQR: interquartile range
OAPR: odds of being affected given a positive result
